# Recent postglacial population expansions may explain a surprising lack of lineage splitting in Arctic meiobenthic flatworms

**DOI:** 10.1186/s12862-026-02511-1

**Published:** 2026-03-21

**Authors:** Joel V. Wernström, Yick Hang Kwan, Tobias R. Vonnahme, Ronnie N. Glud, Andreas Altenburger

**Affiliations:** 1 The Arctic University Museum of Norway, UiT The Arctic University of Norway, Lars Thørings veg 10, Tromsø, 9006 Norway; 2 HADAL & Nordcee, Department of Biology, University of Southern Denmark, Campusvej 55, Odense, 5230 Denmark; 3 Greenland Climate Research Centre, Greenland Institute of Natural Resources, Kivioq 2, Nuuk, 3900 Greenland; 4 Danish Institute for Advanced Study (DIAS), University of Southern Denmark, Campusvej 55, Odense, 5230 Denmark; 5 Department of Ocean and Environmental Sciences, Tokyo University of Marine Science and Technology, 4-5-7 Konan, Minato-ku, Tokyo, 108-8477 Japan

**Keywords:** Dispersal, Flatworms, Haplotype networks, Meiobenthos, Morphology, Phylogeography, Speciation

## Abstract

**Background:**

Meiobenthic metazoans often showcase a puzzling combination of poor dispersal capabilities and wide geographical distributions. Because meiofauna ‘should’ be prone to endemism, the surprising observation of widely distributed morphospecies has spurred substantial interest and explanatory attempts. We place the searchlight on two small flatworm species – *Itaspiella helgolandica* and *Notocaryoplana arctica* – which are particularly restricted in habitat preferences and dispersal capabilities but which occur across the Arctic. Two hypotheses which could account for their wide distributions are explored: (1) populations are genetically divergent but showcase morphological stasis in an instance of cryptic speciation and (2) postglacial population expansion occurred recently enough that genetic drift and local adaptation has not produced divergent allopatric clades yet.

**Results:**

We observed consistent gross and copulatory stylet morphology across local and global scales, confirming the general lack of morphological differences noted by other authors. Our phylogenetic analyses and species delimitation test (bPTP) of partial ribosomal gene sequences (*28 S* and *18 S rDNA*) support the monophyly of each species, with no apparent signs of nascent allopatric lineage splitting. Likewise, haplotype networks do not indicate geography-derived clustering of populations but rather point toward interconnectivity across global and local scales. While our sample sizes and the genetic markers included limit the interpretations of the study, the star-like shapes of the haplotype networks together with negative Tajima’s D and significant, negative Fu’s Fs test values strongly point toward a history of recent population expansions in the studied flatworms.

**Conclusions:**

We find strong support for postglacial population expansions as the most parsimonious explanation for the observed morphological similarities and a weakening of competing hypotheses, e.g. that of widespread cryptic speciation. We note that both species may still be dispersing over long distances e.g. through drifting of egg capsules, and our findings carry important implications for phylogeographical studies of meiobenthic invertebrates and the associated ‘meiofauna paradox’.

**Supplementary Information:**

The online version contains supplementary material available at 10.1186/s12862-026-02511-1.

## Background

Meiobenthic animals are small-sized, inhabit aquatic sediments across the Earth, and often showcase direct development [49]. This reproductive strategy, where offspring begin their lives as miniature adults rather than as a planktonic larvae capable of long-range dispersal, imposes an apparent limitation on meiobenthos dispersal as compared to many other marine invertebrate groups. As large ranges tend to increase speciation rates [47], a fair assumption would be that most meiobenthic species had exceptional potential for endemism [34], and consequently, small ranges. Over time, the surprising record of meiobenthic species distributed across large areas therefore precipitated a vibrant discussion about the possible explanations for this phenomenon, and added up to the formulation of a ‘meiofauna paradox’ which has been a recurring focal point in meiobenthic research [3, 12, 16, 19, 26, 31, 59]. A related concept, the ‘ubiquity theorem’ [22] acknowledges that small organisms often have wide ranges – but in meiobenthic groups, poor dispersal potential still translates to restricted distributions [19].

New insights into the ‘meiofauna paradox’ became available with the advent of phylogeographical and population genetic approaches, which have revealed a vast hidden diversity of cryptic species among the meiobenthos [14, 49, 63]. While masking of genetic variation by morphological stasis is certainly prevalent among meiobenthic taxa, several other evolutionary scenarios, ecological filtering, life-history traits, and palaeogeographical circumstances may also come into play in explaining surprisingly wide distributions [16, 19, 39]. In the Arctic, retracing the recent evolutionary history of the marine fauna is complicated by trans-Arctic dispersal events which have taken place throughout the Pliocene-Pleistocene-Holocene time frame. Repeated postglacial range expansions of marine organisms have led to several large-scale invasion waves with resultant secondary contacts and hybridisation of populations in numerous groups of macroscopic animals [32]. How such cycles of trans-Arctic dispersal and vicariance have affected less dispersal-prone members of the meiobenthos, however, remains mostly unknown.

To explore this, we have sampled specimens of two flatworm species in the proseriate family Otoplanidae Hallez, 1892. We initially became familiar with these species – *Itaspiella helgolandica* [40] and *Notocaryoplana arctica* Steinböck, 1935 – during opportunistic sampling in northern Norway, where they are both common constitutents of littoral meiobenthic faunas. Both species are restricted to complex but marginal habitats – the surf zone of sandy beaches – and lack planktonic larvae capable of long-range dispersal [7, 8, 38, 39]. Within the surf zone, otoplanids are fast-moving meiobenthic top predators which hunt and eat other tiny animals, including copepods, nematodes and other flatworms [20, 50, 61]. Despite their apparent lack of adaptations enabling long-range dispersal, specimens of morphologically consistent otoplanid species have been reported from various locations across the Arctic [6–11, 54, 61]. While hypothetical means of dispersal for otoplanids have been speculated upon, including the spreading of eggs or cysts e.g. by seabirds or drifting [8–10], the explanation for circumpolar distributions in these spatially restricted and dispersal-averse flatworms remains unclear. It is further complicated by the frequently reported affinity of many circumpolar flatworms for brackish water and inability to survive in pure freshwater or at full marine salinities [8–10].

**Fig. 1:**
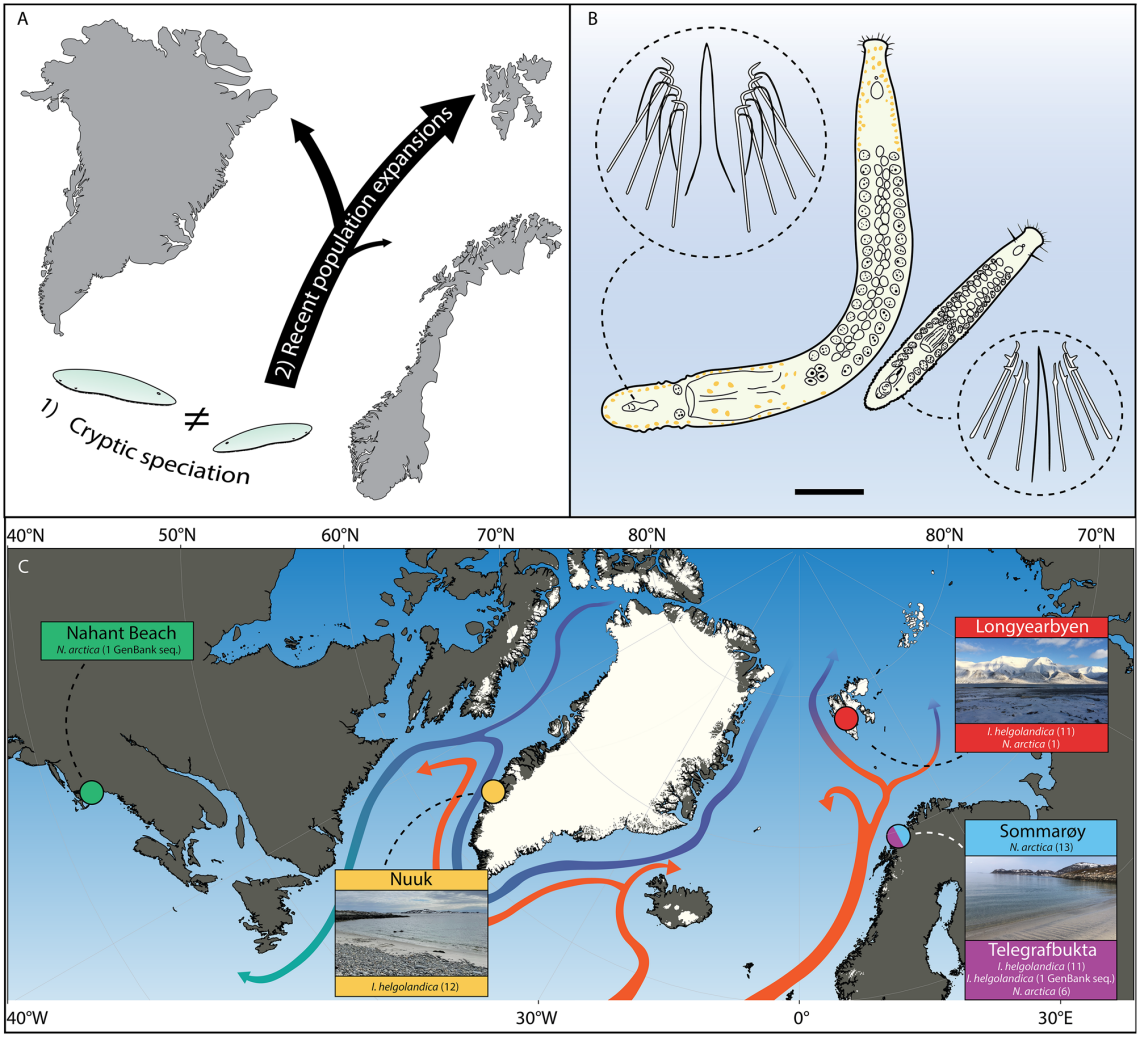
**(A)** Schematic overview of two hypotheses which could explain the apparent wide geographical ranges of the two studied otoplanid flatworm species in the Arctic. **(B)** Outline drawings of *Notocaryoplana arctica* (upper left) and *Itaspiella helgolandica* (lower right) gross morphology and typical appearance of stylet apparatuses after [6, 61]. Body sizes to scale after mean lengths reported by [6]. Scale bar = 500 μm. **(C)** Map of flatworm specimen origins in the Arctic and North Atlantic with major ocean currents after [1, 17, 48]. Colours designate separate sampling sites, with green denoting the origin of a GenBank reference sequence for *N. arctica* included in this study

We explore two hypotheses which can account for the wide ranges (Fig. 1A). These are comprised of (1) that populations represent genetically divergent but morphologically static cryptic allopatric species, possibly as a result of stabilising selection or other evolutionary phenomena which have been attested in the meiobenthos [16] and (2) recent (assumedly postglacial) dispersal events, with gene flow possibly still occurring. Like many otoplanid taxa, the two species studied herein have undergone several taxonomic synonymisations (see e.g. Ax [5], Ax [7], Sopott [56], but are morphologically well-defined on basis of their copulatory stylet apparatus morphologies (Fig. 1B). In *I. helgolandica*, this apparatus consists of a thin, arched stylet tube of 30–35 μm in length surrounded by a bundle of eight needles up to 35 μm in length [61]. In *N. arctica*, it consists of a hairpin-shaped stylet tube of 50–70 μm in length with and 8—14 needles of up to 60 μm in length [6]. Importantly, both species have been reported as morphologically consistent throughout their distributions, aptly summarized by premier authorities on circumpolar flatworms: “*Species common to both sides of the Atlantic show no or only minor morphological differences… the variation between European and Canadian specimens does not exceed the variation found within European populations.”* [9]. To shed light on the processes responsible for the wide distributions of these two otoplanid species, we have collected specimens at beaches across the Arctic, including areas in Greenland, Svalbard, and mainland northern Norway (Fig. 1C).

Based on the collected material, we then acquired and analysed partial sequenes of the* 28S* and* 18S rDNA* genes and subjected them to phylogenetic and population genetic analyses as well as species delimitation testing. In light of th generated phylogeographic data, we then discuss the two competing hypotheses outlined above.

## Methods

### Sampling and morphological study of flatworm specimens

Flatworm specimens were retrieved from marine beaches in Greenland (Nuuk), Svalbard (Longyearbyen), and Norway [62] by collecting surf-zone sand and seawater in a bucket between April and August, 2024. In northern Norway, two locations were sampled – the in-fjord Telegrafbukta beach in Tromsø city, and the island of Sommarøy in the outermost archipelago. In Nuuk and Sommarøy, a sea water salinity measurement was taken using a Kern Optics analog refractometer. Flatworms were extracted from recovered sand by mixing it with a solution of 7% MgCl_2_ in sea water in an Erlenmeyer flask. The flask was turned upside down a couple of times before decanting the aqueous solution with suspended flatworms into a 63 μm sieve. The sieve was placed in a petri dish containing sea water before flatworms were manually picked out, and specimens of each species placed into separate embryo dishes using a glass Pasteur pipette. When practically possible, specimens were starved in clean seawater in a refrigerator for a few days before they were photographed under microscopes or stereo microscopes, depending on what was available at the sampling location. In our examinations, we focused on gross and copulatory stylet apparatus morphology, the latter being widely regarded as the primary character for species identifications in Proseriata [52]. Compression of the worms was needed to visualise the sclerotized stylet apparatus, and we took the following approach: specimen immobilization in a 7% MgCl_2_ solution, transfer to a microscope slide, addition of a cover slip with small feet of beeswax, and compression through careful removal of liquid using a filter paper. For specimens collected in Svalbard, rough seas and engine vibrations precluded detailed documentation of morphological data in the research vessel laboratory, but gross and stylet morphologies were observed by eye. After microscopical documentation, worms were designated with specimen numbers and preserved in individual 3 mL containers filled with absolute, molecular-grade ethanol. Preserved specimens were stored in a -40° C freezer before DNA extraction.

### Extraction and amplification of DNA

In the laboratory, each preserved flatworm specimen was processed individually for DNA extraction. DNA was extracted using DNeasy Blood & Tissue kits (Qiagen) according to the manufacturer’s protocol, except that final elution volume were adjusted to 100 µL to ensure DNA extracts were concentrated enough for polymerase chain reaction (PCR) amplification. The DNA extracts were then kept at 20 °C prior to PCR amplifications. The PCRs were performed with 28S *rDNA* primer pair LSU5 (forward) and LSUD6-3 (reverse) to amplify the 1800 bp ribosomal *28S* D1-D6 fragment while the *18S* A (forward) and 18S B (reverse) primer pair was used to amplify the complete *18S rDNA* gene [53]. A 25 µL PCR reaction mix for each DNA extracts were prepared with 12.5 µL of Hot Start Taq 2X Master Mix (New England Biolabs, UK), 0.5 µL (concentration: 10 pmol/µL) of the forward and reverse primer, 10.5 µL PCR water (ThermoFisher, USA) and 1 µL of template. PCR negatives were performed by replacing the template with PCR water. PCR amplifications were run with the following settings: 30s at 95 °C, 30 cycles: 30s at 95 °C, 60s at 53 °C and 90s at 68 °C, then 5 min at 68 °C for final extension. The PCR products were purified using PureLink PCR Purification Kit (ThermoFisher, USA) according to the manufacturer’s protocol. A gel electrophoresis was conducted with all purified PCR products to ensure the expected product sizes of the gene fragments were obtained. The purified PCR products were then sequenced for both forward and reverse primers, through an external sequencing service (Eurofins, Germany) on a Sanger sequencing platform. Generated sequences were published at the European Nucleotide Archive (ENA) and their provenance is outlined in the specimen table associated with this article (Add. File 1).

### Phylogenetics, species delimitation tests and haplotype network analysis

Bioinformatic analyses were done with software at default settings unless otherwise specified. The forward and reverse raw Sanger sequences obtained from the same flatworm specimen were assembled using CodonCode Aligner v12.0.3. Previously published *18S* and *28S* sequences of related flatworm taxa were downloaded from the NCBI database as reference sequences in our phylogenetic tree construction. Sequences from a wide variety of Otoplanidae taxa were included, but to avoid redundancy in the tree we did not include all the published sequences. To the best of our knowledge, all reference sequences from the two species studied herein were considered. These encompassed a single reported *28S* sequence from *I. helgolandica* reported from Tromsø, Norway (GenBank accession number OR852428) and two derived from *N. arctica* specimens, one of which was reported from Nahant Beach in the United States [35] and the other from Plymouth in the United Kingdom (with GenBank accession number HM026561). The latter was only included in the earliest stages of analysis and was later excluded (for further information, we refer to the Discussion).

Assembled sequences were aligned with the reference sequences using MAFFT v.7 [28, 29] and the alignments were trimmed with trimAl v2.0 (flag: --automated1). The trimmed alignments of all sequences (fragment sizes: 1420 bp for *18S*, 1400 bp for *28S*) were then used in phylogenetic inference with two approaches: Bayesian Inference (BI) and Maximum Likelihood (ML) method. For the ML tree, the GTR + I+R model were used with 1000 bootstrap replicates, and the model used was confirmed with the “Model Finder Plus” (flag: -m MFP) using IQ-TREE 2 v 2.3.6 [41]. For the BI tree, MrBayes v3.2 [25, 46] was used with the GTR + I+R model (settings: mcmcp, nchains = 4, ngen = 10000000, stopval = 0.019). In both *28S* trees, *Yorknia mediterranea* Curini-Galletti, Casu & Scarpa, 2017 (KY320166) was selected as the outgroup. The trees were then visualised with FigTree v1.4.4, and the tree topologies were verified before merging the BI and ML *28S* trees into one. In the analysis of *18 S* sequences, trees are instead presented separately (Supp. File 1). To test the hypothesis of cryptic speciation, we used bPTP [64], a Bayesian implementation of the Poisson Tree Processes model, to delimit species boundaries in *I. helgolandica* and *N. arctica* based on the rooted *28S* ML tree in Newick format. In the analysis, which was carried out on the bPTP web server, we applied 500 000 Markov Chain Monte Carlo (MCMC) generations with the first 10% discarded as burn-in, and verified chain convergence *a posteriori* via the generated MCMC iteration plots.

Prior to haplotype network analysis, all sequences generated in this study were systematically validated with regard to their phylogenetic position. While the *N. arctica* sequence from Plymouth was discarded as possibly misidentified (see Discussion), the sequence from Nahant Beach was included also in the haplotype network. The haplotype networks for *I. helgolandica* (*n* = 34) and *N. arctica* (*n* = 21, including the reference sequence) were visualised using POPART v1.7 [36] with a minimum spanning network algorithm. In addition, Tajima’D and Fu’s Fs population genetic analyses were performed by using the Arlequin v3.5.1.2 software [21].

## Results

### Sampling effort and morphological examination

**Fig. 2:**
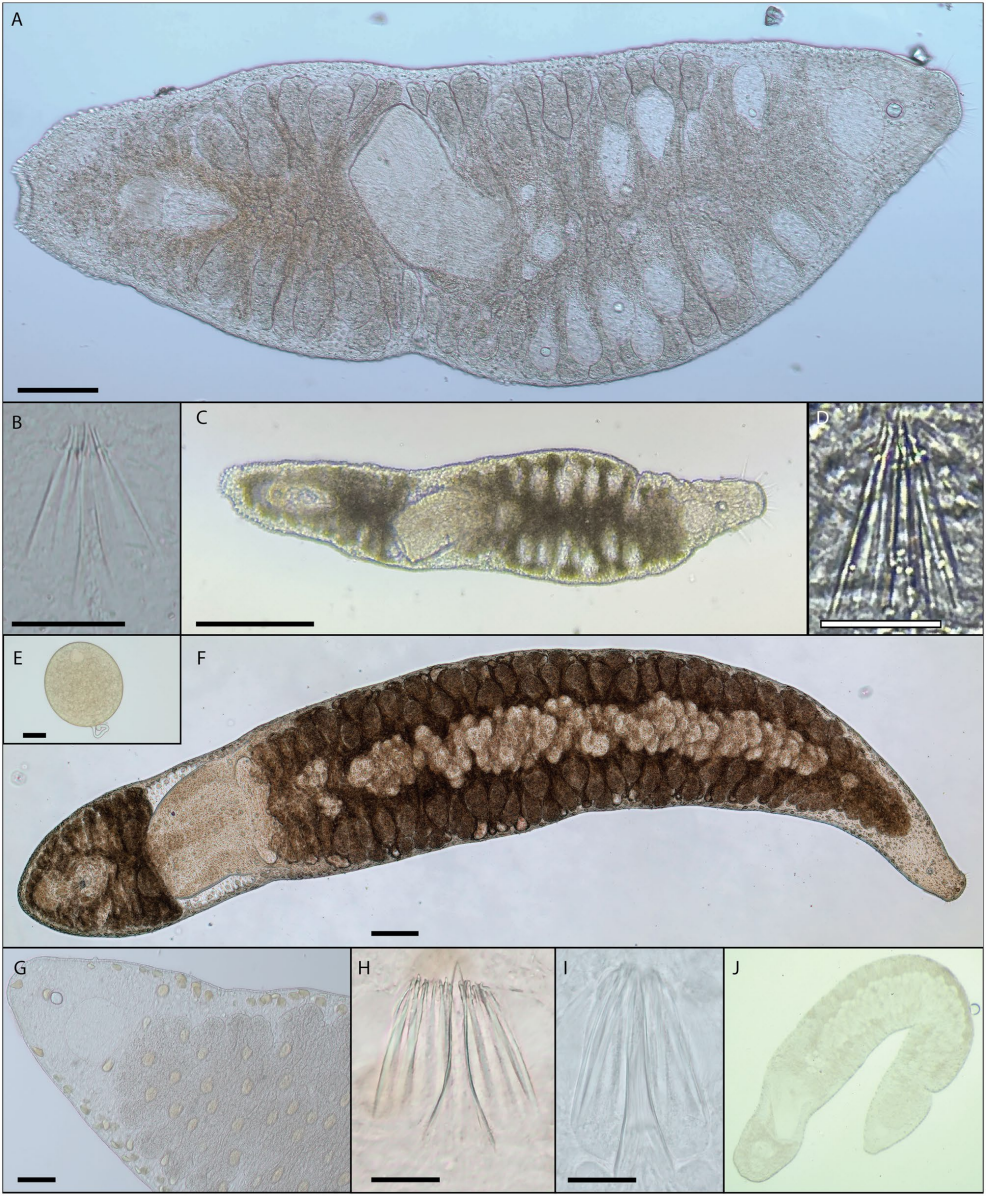
Overview of gross and stylet morphology of otoplanid flatworms *Itaspiella helgolandica* (**A**—**E**) and *Notocaryoplana arctica* (**F**—**I**). **(A)** Composite micrograph of heavily compressed *I. helgolandica* sampled at Telegrafbukta, northern Norway. Scale bar = 100 μm. **(B)** Stylets of specimen from Telegrafbukta. Scale bar = 25 μm. **(C)** Specimen sampled in Nuuk, Greenland. Scale bar = 200 μm. **(D)** Stylets of specimen from Nuuk. Scale bar = 5 μm. **(E)** Focus-stacked micrograph of *I. helgolandica* egg capsule 1 day after deposition. Scale bar = 50 μm. **(F)** Composite micrograph of *N. arctica* sampled in Sommarøy, northern Norway. Scale bar = 200 μm. **(G)** Detail of golden-yellow glands in a specimen sampled in Telegrafbukta. Scale bar = 50 μm. **(H)** Stylets of specimen from Sommarøy. Scale bar = 25 μm. **(I)** Stylets of specimen from Tromsø. Scale bar = 20 μm. **(J)** Unscaled image of specimen sampled in Longyearbyen, Svalbard

We recovered 34 specimens of *I. helgolandica* in Greenland (12), Svalbard (11), and at Telegrafbukta (11) in northern Norway. In addition, we collected 6 specimens of *N. arctica* at Telegrafbukta, 13 in the other northern Norwegian locality, Sommarøy, 1 in Svalbard and one indeterminate otoplanid flatworm in Greenland. Specimens were identified as otoplanid flatworms on basis of their typical gross morphology consisting of a prominent statocyst, a bipartite and ciliated creeping sole, ovoid brain, prominent tufts of head ciliation, a plicate, bulbous pharynx located approximately in the mid-section of the body, a posterior end containing many adhesive papillae and a copulatory bulb with sclerotised needles [13, 61]. Additionally, the characteristic and erratic movement patterns of the worms (sometimes referred to as the ‘otoplanid shake’) were helpful in separating the specimens from other soft-bodied meiobenthic organisms in the samples. Refractometer measurements indicated that seawater salinities were lower in Nuuk (30‰, on the borderline between brackish and euhaline conditions) than in the fully marine site of Sommarøy (39‰, distinctly higher than the expected c. 30—34‰ [43], possibly due to influence of tidal pool water). As for *I. helgolandica* (Fig. 2A—2D), specimens from northern Norway, Greenland and Svalbard all correspond well to the drawings in the original description [40] and display identical morphology of the copulatory stylet apparatus which consists of a thin, arched stylet tube surrounded by a bundle of eight needles with conspicuous apophyses (Fig. 2B and D). The most prominent difference we could identify between northern Norwegian and Greenlandic *I. helgolandica* is that the latter were generally smaller, typically reaching about 800—1000 μm in length instead of the 1000–1200 μm noted for northern Norway [61]. Four congeneric species are known; two from California (*Itaspiella bursituba* and *Itaspiella bodegae* [27], one from Japan (*Itaspiella macrostilifera* [58] and one from freshwater habitats in South America (*Itaspiella parana* [44]. These all differ from *I. helgolandica* in various aspects of their gross morphology and the configuration of their copulatory stylet apparatuses. During storage of live worms, several egg capsules were deposited onto the glass petri dish and attached with a slimy stalk (Fig. 2E), but did not hatch despite weeks-long storage. The larger species *N. arctica* (Fig. 2F—J) was easily identifiable due to its characteristic covering of prominent golden-yellow glands (Fig. 2G), and was morphologically consistent between the two locations in northern Norway and in Svalbard, as well as with the original description [6]. While the stylet tube (c. 50–60 μm) and 8–10 needles (c. 45–50 μm) of our specimens (Fig. 2H and I) were slightly longer than those of the U.S. Pacific coast, they were of largely identical size to those reported from Canada and the German North Sea coast [9]. A single congeneric species (*Notocaryoplana geminofollicularis*) is known exclusively from Japan, and differs from *N. arctica* in several aspects of its morphology including the number of copulatory needles which ranges from 12 to 18 [58]. A full overview with images of our sampled specimens can be found in the associated occurrence dataset uploaded to the Global Biodiversity Information Facility [62].

### Phylogenetic analysis and haplotype networks

**Fig. 3:**
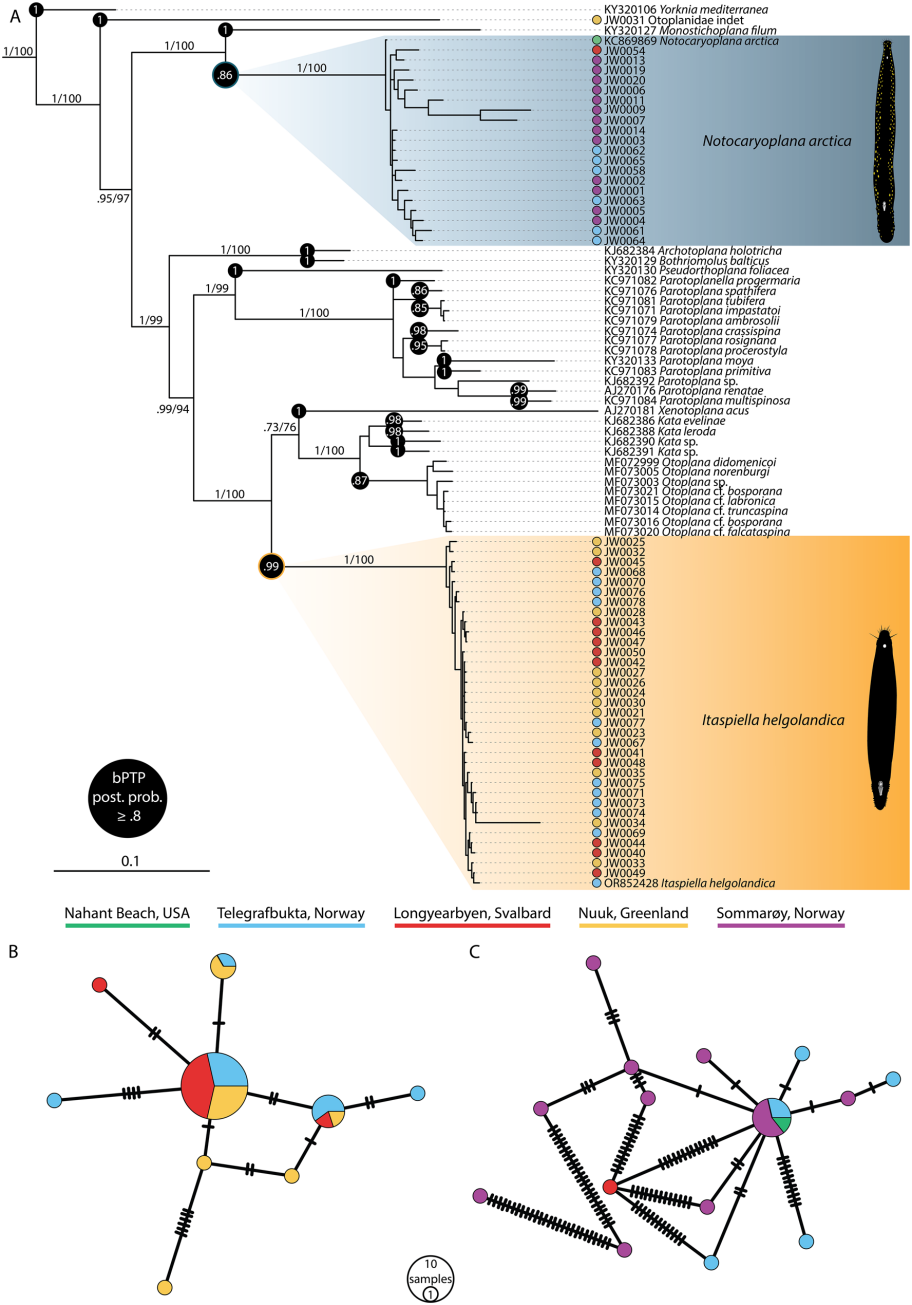
**(A)** Bayesian phylogeny of the Otoplanidae with *Yorknia mediterranea* as outgroup inferred from partial *28S rDNA* sequences. Numbers at select internodes relevant for our conclusions represent posterior probabilities from Bayesian analysis and bootstrap values from maximum likelihood (1000 replicates). Posterior probabilities (≥ 0.8) from the bPTP species delimitation test shown in black circles at nodes. Sequences acquired by us are denoted by their specimen numbers. **(B)** Haplotype network of *Itaspiella helgolandica.* Each traversing black line indicates a nucleotide difference. **(C)** Haplotype network of *Notocaryoplana arctica*

Our phylogenetic analysis and haplotype networks (Fig. 3) indicate that both flatworm species are monophyletic, and show low genetic diversity between specimens across local and global scales. In the phylogenetic analysis of *28S* sequences (Fig. 3A), a similar topology to those of previous authors on otoplanids was observed [53, 61]. We did not observe clear lineage splitting attributable to geographical structuring in the otherwise well-defined *I. helgolandica* and *N. arctica* clades. Instead, sequences from worms sampled far apart often appear as sister groups, in a pattern which is mirrored by our *18S* phylogenies (Add. File 1). Posterior probability and bootstrap support was generally high for most nodes in both trees, including intraspecific nodes. While the long branch lengths of some specimens indicate greater amounts of divergence for certain sequences, the bPTP species delimitation test unambiguously indicates that the two species are valid and that they should not be split further. This result is corroborated by the haplotype networks, which generally indicated few mutational differences across populations (Fig. 3B and C). The *28S rDNA*networks featured a central abundant haplotype which was shared by many specimens from different locations. In the *Itaspiella helgolandica* gene network, we recovered three shared haplotypes in the middle which contained the greatest number of individuals (21). Other haplotypes in the same network showed low mutational differences: for instance, individuals with a sequence difference of 1/2 bp, which divided over a sequence of 1400 bp gives a difference of only 0.0014%. In the *N. arctica* gene network, we observed a similar pattern but with comparatively greater genetic variation in the Sommarøy population. Furthermore, our specimens and the reference sequence obtained from Nahant Beach in the United States – comparatively far away from Svalbard and northern Norway –do not show clearsigns of geographical isolation on this gene fragment. While some specimens of *N. arctica* show more nucleotide differences than others, the most distant specimens are from the same beach in Sommarøy, which indicates that intraspecific variation is larger within sites than between sites. It should be noted, however, that there are clear differences in the number of mutational steps between species and many unique haplotypes. Consequently, *N. arctica* shows more mutational differences among haplotypes than *I. helgolandica*, even though these do not form distinct clades in the tree.

Our Tajimas’ D and Fu’s Fs tests to assess deviations from neutral expectations resulted in negative values for all three studied populations (*n* > 10) of *I. helgolandica* (Table 1).

**Table 1:** Tajima’s D test and Fu’s Fs test summary statistics for *28 S rDNA* sequences derived from three populations of *Itaspiella helgolandica*

Population	No. of individuals	Tajima’s D	Tajima’s D *p*-value	Fu’s Fs	Fu’s Fs *p*-value
Telegrafbukta	12	-0.479	0.346	-9.7564	0
Longyearbyen	11	-1.424	0.095	-16.945	0
Nuuk	12	-1.997	0.004	-4.754	0.016

While it should be noted that the *28S* gene is generally conserved, with low mutation rate and evolutionary neutrality, the output implies that past evolutionary scenarios such as purifying selection or recent population expansions could have occurred. The Tajima’s D and Fu’s Fs tests for *N. arctica* (Add. File 1) were affected by the marked difference in sample sizes, where single sequences derived from Longyearbyen and Nahant Beach were compared to 13 individuals from Telegrafbukta, 6 from Sommarøy, and specimens *n* < 5 for other populations, precluding meaningful neutrality test results.

## Discussion

### Circumpolar otoplanid flatworms showcase monophyly, few morphological differences and lack of lineage splitting

Flatworm specimens determined to the same species were morphologically consistent across widely differing geographical locations, a finding which corroborates the observations of numerous earlier studies [7–10, 61]. Minor morphological variation, such as the comparatively smaller body size of *I. helgolandica* specimens in Nuuk compared to northern Norway, are interesting and could indicate nascent local adaptation, but do not negate our assessment that the current classification into morphospecies appears warranted.

The classification of *I. helgolandica* and *N. arctica* as coherent species is further supported by the fact that both taxa formed monophyletic clades with high branch supporting scores in our *28S* phylogeny, with the *18S* tree showing a very similar topology (Add. File 1). Monophyly of these two species, and the lack of geographical structure in the tree, indicates that underlying populations have not yet undergone allopatric lineage splitting. It should be noted that the phylogenetic topology may imply some intraspecies segregation due to the comparatively long branch lengths retrieved for some specimens, but this may also reflect e.g. the multi-copy and intragenomic heterogeneity characteristics of the *28S* gene, especially considering the fact that branch length patterns differ between the *28S* and *18S* tree. While the possibility remains that these particular specimens represent putative cryptic species, the respective lack of observed morphological differences and of geographical structure in the dataset warrants a cautious interpretation to avoid oversplitting [57]. Because the specimens in question are bracketed by other specimens from the same locations, convincing evidence of genetic isolation would be required for establishing species status [45]. Additionally, the bPTP species delimitation test we performed indicates that the two species are valid and does not support the presence of any cryptic species within them. For *I. helgolandica*, the highest bPTP posterior probability support for an internal node representing a distinct species was 0.01, while for *N. arctica* the corresponding number was 0.14 (Add. File 1). Consequently, the hypothesis of widespread crypticism is substantially weakened.

As for the haplotype networks, neither one clearly indicates geographical clustering of specimens. Sampling locations such as Sommarøy, which is located in the outer archipelago, could be particularly exposed to dispersal events which bring new haplotypes into the site resulting in a highly mixed gene pool at the local scale. Based on the *N. arctica* gene network, it could be argued that the most dissimilar sequences may be derived from nonconspecific specimens which belong to closely related (and possibly undescribed) taxa for which there are currently no reference sequences. However, our morphological examination and phylogenetic analyses generally do not support such an interpretation since they indicate monophyly. The sister-group relationship of US-derived reference sequence labelled as *Notocaryoplana arctica* to our own specimens is interesting, as it points towards a possible split between continental North American and European-Greenlandic lineages. However, in the *18S* gene tree this North American specimen showcased the smallest number of mutational steps as compared to a central haplotype from Sommarøy (Add. File 1), so the results may also reflect incomplete lineage sorting with respect to the *28S* gene. As the Nahant Beach specimen was not sampled by us, whether it differed in morphological characters remains unknown, but is unlikely based on the noted similarities of specimens from North America and Europe [9]. Conversely, during early stages of phylogenetic analysis we retrieved the other existing reference sequence for *N. arctica*, derived from Plymouth in the United Kingdom, as a distantly related sister group to the clade formed by *N. arctica* and *Monostichoplana filum* [40]. We therefore concluded that the specimen in question may have been misidentified, and likely represents a closely related, possibly undescribed taxon. While it would have been informative to include sequence data of congeneric species of *Itaspiella* and *Notocaryoplana* from other parts of the world in our analyses, we did not have the opportunity to sample them and were restricted to morphological comparisons with published accounts. While their classification as different species seems warranted on morphological grounds, the addition of molecular data for those taxa could provide further nuance in the future.

In sum, our analyses do not show signs of lineage splitting attributable to geographical structuring, and like previous authors we did not observe clear intraspecific morphological differences which would warrant species-level reclassifications. Beyond long branch lengths for some specimens, we consequently recovered no evidence for the masking of genetic variation by morphological stasis which has been hinted at in previous studies [61],. Our combination of morphological and molecular analyses therefore provides substantial evidence against hypothesis 1) concerning cryptic speciation as an explanation for the circumpolar distributions of these dispersal-averse flatworms.

### Postglacial cycles of dispersal throughout the Arctic offer the highest explanatory power

On the contrary, the evidence we report strongly supports the case of hypothesis 2), that the morphological (and with our study, molecular) similarity is the result of recent (postglacial) cycles of dispersal. For instance, both of the *28S* haplotype networks have star-like shapes, which are often the result of a recent and rapid expansion scenario [4, 55]. Likewise, the negative Tajima’s D and significant, negative Fu’s Fs values (Table 1 & Add. File 1) fit the signature of a recent and rapid population expansion. This could also explain the number of unique haplotypes observed in both gene networks. Yet, the overall Tajima’s D values for all populations were less than − 2, despite being significant for the Nuuk population of *I. helgolandica*, which implies no excess of rare haplotypes present in these populations and potentially reflects no cryptic speciation which can be observed through the gene fragments studied herein. It should be noted that our sampling sizes are fairly uneven and for some locations rather small, which somewhat limits the conclusions which can be drawn from these statistical tests [33]. While a larger number of specimens would obviously have been preferable, the challenges of finding and identifying these highly sensitive microscopic flatworms in the field limited the number which could be collected, and our work represents most comprehensive of its kind on otoplanid flatworms in the Arctic. Otoplanid flatworm invasion waves would have mirrored the trans-Arctic dispersal events with resultant secondary contacts and hybridisation which have taken place among marine macrofauna since the Pliocene [30, 32], with similar scenarios having been hypothesised for other flatworm taxa [15]. Considering the strong evidence which supports a recent history of range expansions in Arctic macrofauna, it does however seem reasonable to assume that the regional meiobenthos has followed a similar eco-evolutionary trajectory. Whether the time elapsed since these mixing events occurred is sufficient or too short for allopatric lineage splitting to have begun is difficult to determine. Little is known about otoplanid longevity, but using the median 29.6 weeks-long lifespan of the ecologically similar, meiobenthic flatworm *Macrostomum lignano* Ladurner, Schärer, Salvenmoser & Rieger, 2005 as a proxy [42] allows for a rough estimate of c. 19 000—23 000 generations passed since the Bering Strait reopened 13 000—11000 Kya after the last glacial maximum [32]. Assuming that the most recent meiobenthic invasion waves occurred around that time and that selective pressures promotive of cladogenesis were present, means that enough time could theoretically have passed for Arctic otoplanids to diverge into locally adapted and distinct lineages [24]. However, molecular clock data (in lieu of a flatworm fossil record) show that many species-level splits in the Otoplanidae are older than at least 900 Kyr [51], indicating that local adaptation and allopatric lineage splitting is generally a slow process in these flatworms. In lieu of strong, local selective pressures, it is therefore reasonable to assume that postglacial population expansions happened too recently for speciation to occur in these flatworm populations on a pan-Arctic scale. Our impression of the surf-zone habitats we have sampled at are that they appear fairly similar in terms of abiotic factors, and may lack obvious sources of strongly differential selective pressures which could drive rapid speciation. It should be noted that other evolutionary phenomena such as purifying selection (which certainly contributes to the tendency of the *18S* and *28S* genes to remain relatively conserved) could also give rise to patterns similar to those from recent population expansions the ones we observed. However, we see little reason to believe that purifying selection acting on these genes is the shaping force behind the observed patterns. While including a larger number of more variable genes into the analyses would have been ideal, molecular work on microscopic proseriates is often tricky, and in lieu of other reliable PCR primers, *18S* and *28S* are the most common markers relied upon [18, 53, 61]. While we are not aware of any other phylogeographic studies on the species studied herein to which our results could be directly compared, similar studies of proseriate flatworms have been carried out in other parts of the world. For instance, partial sequences of the *28S rDNA* gene were recently used to identify cryptic species in the otoplanid genus *Kata*, with the authors noting that some gene flow may occur across distant locations based on the observation of shared haplotypes among *Kata* populations [18].

Whether postglacial population expansions occurred once, e.g. along the frontiers of retreating glaciers, or are still ongoing is more difficult to determine based on our data. We are sceptical of some previous speculative dispersal pathways which have been entertained by other authors, such as bird-mediated migration or spread through continental drift [9] because these means of dispersal would either be too irregular or too slow to explain our reported levels of genetic similarity and lack of lineage splitting. However, continuous cross-oceanic dispersal through long-range drifting of cysts or egg capsules (Fig. 2E) e.g. attached to macroalgae, ice or other floating substrates are, in our view, a viable explanation for how otoplanid flatworms may have been able to spread across the Arctic both historically and today. In support of this, we observed egg capsules remaining intact and apparently alive during weeks-long refrigerated storage in sea water, indicating that they are capable of long-range passive dispersal before the onset of hatching. It is well known that small-sized marine invertebrates can hitchhike with ice [37]. Drifting experiments have also demonstrated that floating macroalgae are capable of intercontinental travel within reasonable time spans (e.g. completing a journey between Greenland and Newfoundland in 197 days [2]. Additionally, we recovered specimens of the brackish water (10—16‰tolerance [10], flatworm *I. helgolandica* at full marine salinities in Nuuk (which have also been attested in other studies [60], showing a large physiological niche and potential for dispersal. Meanwhile, *N. arctica* were present at Sommarøy, located in an area of fully marine salinities ranging between 30–34‰ [23, 43]. These observations of a very wide ecological salinity-niche spanning from brackish to fully marine conditions could in themselves point to a higher potential for cross-oceanic dispersal in these species than has been assumed previously, and if egg capsules can survive weeks-long transport, dispersal across our studied areas is conceivable based on the major ocean currents of the north Atlantic and Arctic (Fig. 1C). As such, the ‘meiofauna paradox’ in Arctic flatworms may at least partially be derived from an underappreciation of their dispersal capabilities.

In sum, we find support for the hypothesis of postglacial population expansions, where migration could occur through e.g. drifting of egg capsules. However, our sampling size and the fact that we examine only two ribosomal genes makes our data insufficient for more in-depth population genetic analysis and precludes reliable identification of the actual genetic flow or migration models. Hence, we cannot confidently demonstrate current connectivity between the populations, and the limitations of our approach necessitates a careful consideration of the results. An approach which accounts for this along with the retracing of past meiobenthic invasion waves (e.g. through sedaDNA analysis) into the Arctic would be interesting venues for future research.

## Conclusions

Based on the monophyly and lack of geographical structure in *I. helgolandica* and *N. arctica* populations from across the Arctic, our evidence weakens the hypothesis of widespread genetic diversity masked by morphological stasis as an explanation for the wide distributions of the flatworms studied herein. Rather, we conclude that representatives of neither taxon have diverged into distinct species since the postglacial expansion of their ranges occurred, and note that they may still be dispersing over long distances. By suggesting that we tend to understimate the dispersal capabilities of meiobenthic invertebrates, our findings carry important implications for further phylogeographical studies and for the the ‘meiofauna paradox’.

## Supplementary Information

Below is the link to the electronic supplementary material.

**Supplementary Material 1: Additional File 1.** Fig. A1. *18 S rDNA* phylogeny and gene networks of *Itaspiella helgolandica* and *Notocaryoplana arctica*. Tab. A1. Tajima’s D test and Fu’s FS test summary statistics for *28 S rDNA* sequences derived from four populations of *Notocaryoplana arctica*. Tab. A2. Specimen table with species, locality, ENA nucleotide accession numbers and direct links to GBIF images. Fig. A2. The acquired partial *28 S* maximum likelihood phylogeny of the Otoplanidae annotated with bPTP posterior probabilities for all nodes.

## Data Availability

A comprehensive specimen table with ID: s and their associated information is found in Additional File 1. The occurrence dataset containing image data generated and analysed during the current study are available in the Global Biodiversity Information Facility (GBIF) repository and can be retrieved at https://doi.org/10.15468/62n2u7. Generated gene sequences are available in the European Nucleotide Archive (ENA) at the European Bioinformatics Institute (EMBL-EBI) repository, under project accession number PRJEB96238.
