# Supplementary material for: Recent postglacial population expansions may explain a surprising lack of lineage splitting in Arctic meiobenthic flatworms
Source: BMC Ecol Evol. 2026 Mar 21;26:27. doi: 10.1186/s12862-026-02511-1 (PMC13011653; doi:10.1186/s12862-026-02511-1)
Supplement: Supplementary file 1 — Supplementary Material 1: Additional File 1. Fig. A1. 18 S rDNA phylogeny and gene networks of Itaspiella helgolandica and Notocaryoplana arctica. Tab. A1. Tajima’s D test and Fu’s FS test summary statistics for 28 S rDNA sequences derived from four populations of Notocaryoplana arctica. Tab. A2. Specimen table with species, locality, ENA nucleotide accession numbers and direct links to GBIF images. Fig. A2. The acquired partial 28 S maximum likelihood phylogeny of the Otoplanidae annotated with bPTP posterior probabilities for all nodes. [file 12862_2026_2511_MOESM1_ESM.docx]

**Recent postglacial population expansions may explain a surprising lack of lineage splitting in Arctic meiobenthic flatworms. BMC Ecology and Evolution. 2026. Additional file 1.**


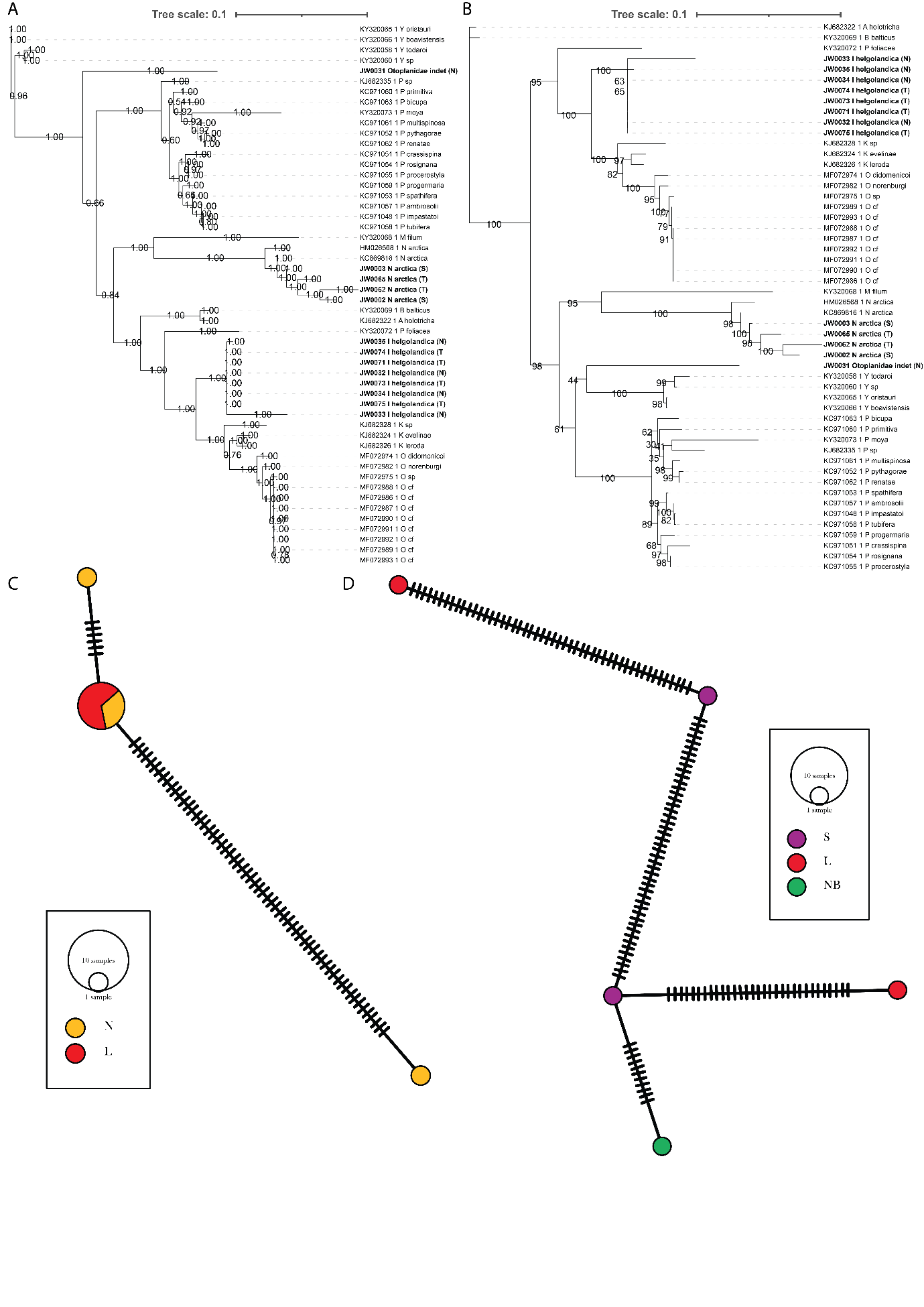


**Additional figure 1. A.** Bayesian phylogeny of the Otoplanidae inferred from partial 18S rDNA sequences, those which acquired herein are denoted by bold text. S = Sommarøy, L = Longyearbyen, NB = Nahant Beach, N = Nuuk, T = Telegrafbukta. **B.** Maximum likelihood phylogeny of the Otoplanidae inferred from 18S rDNA sequences. **C.** Haplotype network of Itaspiella helgolandica 18S sequences. **D.** Haplotype network of Notocaryoplana arctica 18S sequences.

**Additional table 1.** Tajima’s D test and Fu’s FS test summary statistics for 28S rDNA sequences derived from four populations of Notocaryoplana arctica.

| Population | No. of individuals | Tajima’s D | Tajima’s D p-value | Fu’s FS | Fu’s FS p-value |
| --- | --- | --- | --- | --- | --- |
| Telegrafbukta | 13 | -1.02048 | 0.154 | -2.96855 | 0.077 |
| Sommarøy | 6 | -1.48571 | 0.012 | -1.81313 | 0.049 |
| Longyearbyen | 1 | 0 | 1 | 0 | / |
| Nahant Beach | 1 | 0 | 1 | 0 | / |

**Additional table 2.** Specimen table with species, locality, *28S rDNA* accession numbers in ENA and GBIF identifiers providing direct links to images.

| **Specimen ID** | **Species** | **Locality** | **ENA *28S* accession number** | **GBIF identifier** |
| --- | --- | --- | --- | --- |
| JW0023 | *Itaspiella helgolandica* | Nuuk, Greenland | SAMEA119530892 | https://storage.gbif-no.sigma2.no/img/Notocaryoplana/JW0023-1.jpeg |
| JW0067 | *Itaspiella helgolandica* | Telegrafbukta, Norway | SAMEA119530908 | https://storage.gbif-no.sigma2.no/img/Notocaryoplana/JW0067.E-1.png |
| JW0046 | *Itaspiella helgolandica* | Longyearbyen, Svalbard | SAMEA119530893 | https://storage.gbif-no.sigma2.no/img/Notocaryoplana/JW0046.E-1.jpg |
| JW0047 | *Itaspiella helgolandica* | Longyearbyen, Svalbard | - | https://storage.gbif-no.sigma2.no/img/Notocaryoplana/JW0047.E-2.jpg |
| JW0043 | *Itaspiella helgolandica* | Longyearbyen, Svalbard | SAMEA119530895 | https://storage.gbif-no.sigma2.no/img/Notocaryoplana/JW0043.E-1.jpg |
| JW0077 | *Itaspiella helgolandica* | Telegrafbukta, Norway | SAMEA119530896 | https://storage.gbif-no.sigma2.no/img/Notocaryoplana/JW0077.E-3.png |
| JW0049 | *Itaspiella helgolandica* | Longyearbyen, Svalbard | - | https://storage.gbif-no.sigma2.no/img/Notocaryoplana/JW0049.E-1.jpg |
| JW0040 | *Itaspiella helgolandica* | Longyearbyen, Svalbard | SAMEA119530897 | https://storage.gbif-no.sigma2.no/img/Notocaryoplana/JW0040.E-2.jpg |
| JW0026 | *Itaspiella helgolandica* | Nuuk, Greenland | SAMEA119530907 | https://storage.gbif-no.sigma2.no/img/Notocaryoplana/JW0026-7.jpeg |
| JW0044 | *Itaspiella helgolandica* | Longyearbyen, Svalbard | SAMEA119530898 | https://storage.gbif-no.sigma2.no/img/Notocaryoplana/JW0044.E-1.jpg |
| JW0069 | *Itaspiella helgolandica* | Telegrafbukta, Norway | SAMEA119530899 | https://storage.gbif-no.sigma2.no/img/Notocaryoplana/JW0069.E-1.png |
| JW0027 | *Itaspiella helgolandica* | Nuuk, Greenland | SAMEA119530900 | https://storage.gbif-no.sigma2.no/img/Notocaryoplana/JW0027-1.jpeg |
| JW0028 | *Itaspiella helgolandica* | Nuuk, Greenland | - | https://storage.gbif-no.sigma2.no/img/Notocaryoplana/JW0028-1.jpeg |
| JW0021 | *Itaspiella helgolandica* | Nuuk, Greenland | SAMEA119530902 | https://storage.gbif-no.sigma2.no/img/Notocaryoplana/JW0021-3.jpeg |
| JW0030 | *Itaspiella helgolandica* | Nuuk, Greenland | - | https://storage.gbif-no.sigma2.no/img/Notocaryoplana/JW0030-1.jpeg |
| JW0041 | *Itaspiella helgolandica* | Longyearbyen, Svalbard | SAMEA119530904 | https://storage.gbif-no.sigma2.no/img/Notocaryoplana/JW0041.E-2.jpg |
| JW0048 | *Itaspiella helgolandica* | Longyearbyen, Svalbard | SAMEA119530905 | https://storage.gbif-no.sigma2.no/img/Notocaryoplana/JW0048.E-1.jpg |
| JW0024 | *Itaspiella helgolandica* | Nuuk, Greenland | SAMEA119530906 | https://storage.gbif-no.sigma2.no/img/Notocaryoplana/JW0024-1.jpeg |
| JW0034 | *Itaspiella helgolandica* | Nuuk, Greenland | SAMEA119530909 | https://storage.gbif-no.sigma2.no/img/Notocaryoplana/JW0034-1.jpeg |
| JW0073 | *Itaspiella helgolandica* | Telegrafbukta, Norway | SAMEA119530910 | https://storage.gbif-no.sigma2.no/img/Notocaryoplana/JW0073.E-1.png |
| JW0074 | *Itaspiella helgolandica* | Telegrafbukta, Norway | SAMEA119530911 | https://storage.gbif-no.sigma2.no/img/Notocaryoplana/JW0074.E-1.png |
| JW0075 | *Itaspiella helgolandica* | Telegrafbukta, Norway | SAMEA119530912 | https://storage.gbif-no.sigma2.no/img/Notocaryoplana/JW0075.E-1.png |
| JW0071 | *Itaspiella helgolandica* | Telegrafbukta, Norway | SAMEA119530913 | https://storage.gbif-no.sigma2.no/img/Notocaryoplana/JW0071.E-5.png |
| JW0033 | *Itaspiella helgolandica* | Nuuk, Greenland | SAMEA119530915 | https://storage.gbif-no.sigma2.no/img/Notocaryoplana/JW0033-1.jpeg |
| JW0035 | *Itaspiella helgolandica* | Nuuk, Greenland | SAMEA119530914 | https://storage.gbif-no.sigma2.no/img/Notocaryoplana/JW0035-3.jpeg |
| JW0032 | *Itaspiella helgolandica* | Nuuk, Greenland | SAMEA119530916 | https://storage.gbif-no.sigma2.no/img/Notocaryoplana/JW0032-1.jpeg |
| JW0068 | *Itaspiella helgolandica* | Telegrafbukta, Norway | SAMEA119530917 | https://storage.gbif-no.sigma2.no/img/Notocaryoplana/JW0068.E-2.png |
| JW0045 | *Itaspiella helgolandica* | Longyearbyen, Svalbard | SAMEA119530919 | https://storage.gbif-no.sigma2.no/img/Notocaryoplana/JW0045.E-1.jpg |
| JW0025 | *Itaspiella helgolandica* | Nuuk, Greenland | SAMEA119530921 | https://storage.gbif-no.sigma2.no/img/Notocaryoplana/JW0025-1.jpeg |
| JW0070 | *Itaspiella helgolandica* | Telegrafbukta, Norway | SAMEA119530920 | https://storage.gbif-no.sigma2.no/img/Notocaryoplana/JW0070.E-4.png |
| JW0076 | *Itaspiella helgolandica* | Telegrafbukta, Norway | SAMEA119530922 | https://storage.gbif-no.sigma2.no/img/Notocaryoplana/JW0076.E-6.png |
| JW0078 | *Itaspiella helgolandica* | Telegrafbukta, Norway | SAMEA119530918 | https://storage.gbif-no.sigma2.no/img/Notocaryoplana/JW0078.png |
| JW0050 | *Itaspiella helgolandica* | Longyearbyen, Svalbard | - | https://storage.gbif-no.sigma2.no/img/Notocaryoplana/JW0050.E-2.jpg |
| JW0042 | *Itaspiella helgolandica* | Longyearbyen, Svalbard | SAMEA119530924 | https://storage.gbif-no.sigma2.no/img/Notocaryoplana/JW0042.E-2.jpg |
| JW0061 | *Notocaryoplana arctica* | Telegrafbukta, Norway | SAMEA119530926 | https://storage.gbif-no.sigma2.no/img/Notocaryoplana/JW0061.E-2.png |
| JW0004 | *Notocaryoplana arctica* | Sommarøy, Norway | SAMEA119530929 | https://storage.gbif-no.sigma2.no/img/Notocaryoplana/JW0004-11.png |
| JW0005 | *Notocaryoplana arctica* | Sommarøy, Norway | SAMEA119530927 | https://storage.gbif-no.sigma2.no/img/Notocaryoplana/JW0005-17.png |
| JW0064 | *Notocaryoplana arctica* | Telegrafbukta, Norway | SAMEA119530928 | https://storage.gbif-no.sigma2.no/img/Notocaryoplana/JW0064.E-2.png |
| JW0063 | *Notocaryoplana arctica* | Telegrafbukta, Norway | SAMEA119530930 | https://storage.gbif-no.sigma2.no/img/Notocaryoplana/JW0063.E-6.png |
| JW0001 | *Notocaryoplana arctica* | Sommarøy, Norway | SAMEA119530931 | https://storage.gbif-no.sigma2.no/img/Notocaryoplana/JW0001-2.png |
| JW0062 | *Notocaryoplana arctica* | Telegrafbukta, Norway | SAMEA119530932 | https://storage.gbif-no.sigma2.no/img/Notocaryoplana/JW0062.E-8.png |
| JW0003 | *Notocaryoplana arctica* | Sommarøy, Norway | SAMEA119530933 | https://storage.gbif-no.sigma2.no/img/Notocaryoplana/JW0003-09.png |
| JW0065 | *Notocaryoplana arctica* | Telegrafbukta, Norway | SAMEA119530934 | https://storage.gbif-no.sigma2.no/img/Notocaryoplana/JW0065.E-1.png |
| JW0019 | *Notocaryoplana arctica* | Sommarøy, Norway | SAMEA119530936 | https://storage.gbif-no.sigma2.no/img/Notocaryoplana/JW00019-1.png |
| JW0006 | *Notocaryoplana arctica* | Sommarøy, Norway | SAMEA119530941 | https://storage.gbif-no.sigma2.no/img/Notocaryoplana/JW0006-7.png |
| JW0058 | *Notocaryoplana arctica* | Telegrafbukta, Norway | SAMEA119530937 | https://storage.gbif-no.sigma2.no/img/Notocaryoplana/JW0058.E-9-composite.png |
| JW0054 | *Notocaryoplana arctica* | Longyearbyen, Svalbard | SAMEA119530939 | https://storage.gbif-no.sigma2.no/img/Notocaryoplana/JW0054.E-1.jpg |
| JW0014 | *Notocaryoplana arctica* | Sommarøy, Norway | SAMEA119530938 | https://storage.gbif-no.sigma2.no/img/Notocaryoplana/JW00014-2.png |
| JW0013 | *Notocaryoplana arctica* | Sommarøy, Norway | SAMEA119530940 | https://storage.gbif-no.sigma2.no/img/Notocaryoplana/JW00013-2.png |
| JW0007 | *Notocaryoplana arctica* | Sommarøy, Norway | SAMEA119530942 | https://storage.gbif-no.sigma2.no/img/Notocaryoplana/JW0007-12.png |
| JW0009 | *Notocaryoplana arctica* | Sommarøy, Norway | SAMEA119530943 | https://storage.gbif-no.sigma2.no/img/Notocaryoplana/JW0009-4.png |
| JW0011 | *Notocaryoplana arctica* | Sommarøy, Norway | SAMEA119530944 | https://storage.gbif-no.sigma2.no/img/Notocaryoplana/JW00011-1.png |
| JW0020 | *Notocaryoplana arctica* | Sommarøy, Norway | SAMEA119530945 | https://storage.gbif-no.sigma2.no/img/Notocaryoplana/JW00020-2.png |
| JW0002 | *Notocaryoplana arctica* | Sommarøy, Norway | SAMEA119530935 | https://storage.gbif-no.sigma2.no/img/Notocaryoplana/JW0002.png |
| JW0031 | Otoplanidae | Nuuk, Greenland | - | https://storage.gbif-no.sigma2.no/img/Notocaryoplana/JW0031-2.jpeg |

**
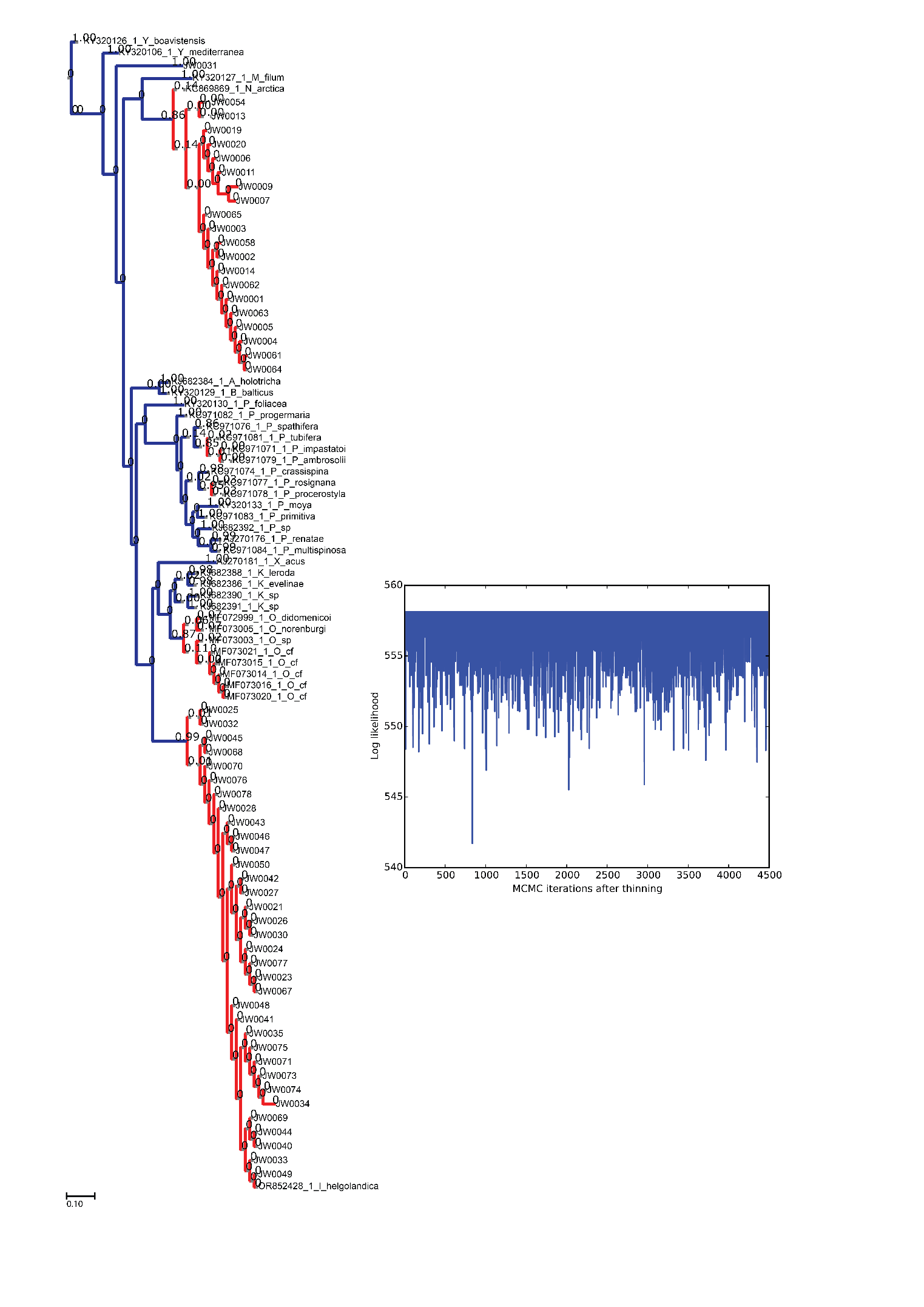
**

**Additional figure 2. A.** ML phylogeny of the Otoplanidae inferred from partial 28S rDNA sequences with annotation consisting of the bPTP posterior probability values for each node and the likelihood trace plot showing chain convergence displayed as an insert.
